# Arylvinylpiperazine Amides, a New Class of Potent Inhibitors Targeting QcrB of Mycobacterium tuberculosis

**DOI:** 10.1128/mBio.01276-18

**Published:** 2018-10-09

**Authors:** Caroline S. Foo, Andréanne Lupien, Maryline Kienle, Anthony Vocat, Andrej Benjak, Raphael Sommer, Dirk A. Lamprecht, Adrie J. C. Steyn, Kevin Pethe, Jérémie Piton, Karl-Heinz Altmann, Stewart T. Cole

**Affiliations:** a Global Health Institute, École Polytechnique Fédérale de Lausanne, Lausanne, Switzerland; b Department of Chemistry and Applied Biosciences, Institute of Pharmaceutical Sciences, ETH Zürich, Zurich, Switzerland; c Africa Health Research Institute, Durban, South Africa; d Department of Microbiology, University of Alabama at Birmingham, Birmingham, Alabama, USA; e Lee Kong Chian School of Medicine and School of Biological Sciences, Nanyang Technological University, Singapore; Sequella, Inc.

**Keywords:** QcrB inhibitor, cytochrome *bc*_1_ oxidase, cytochrome *bd* oxidase, mycobacterial diseases, mycobacterial respiration, tuberculosis

## Abstract

New drugs are needed to control the current tuberculosis (TB) pandemic caused by infection with Mycobacterium tuberculosis. We report here on our work with AX-35, an arylvinylpiperazine amide, and four related analogs, which are potent antitubercular agents *in vitro*. All five compounds showed good activity against M. tuberculosis *in vitro* and in infected THP-1 macrophages, while displaying only mild cytotoxicity. Isolation and characterization of M. tuberculosis-resistant mutants to the arylvinylpiperazine amide derivative AX-35 revealed mutations in the *qcrB* gene encoding a subunit of cytochrome *bc*_1_ oxidase, one of two terminal oxidases of the electron transport chain. Cross-resistance studies, allelic exchange, transcriptomic analyses, and bioenergetic flux assays provided conclusive evidence that the cytochrome *bc*_1_*-aa*_3_ is the target of AX-35, although the compound appears to interact differently with the quinol binding pocket compared to previous QcrB inhibitors. The transcriptomic and bioenergetic profiles of M. tuberculosis treated with AX-35 were similar to those generated by other cytochrome *bc*_1_ oxidase inhibitors, including the compensatory role of the alternate terminal oxidase cytochrome *bd* in respiratory adaptation. In the absence of cytochrome *bd* oxidase, AX-35 was bactericidal against M. tuberculosis. Finally, AX-35 and its analogs were active in an acute mouse model of TB infection, with two analogs displaying improved activity over the parent compound. Our findings will guide future lead optimization to produce a drug candidate for the treatment of TB and other mycobacterial diseases, including Buruli ulcer and leprosy.

## Abstract (precis)

New drugs against Mycobacterium tuberculosis are urgently needed to deal with the current global TB pandemic. We report here on the discovery of a series of arylvinylpiperazine amides (AX-35 to AX-39) that represent a promising new family of compounds with potent *in vitro* and *in vivo* activities against M. tuberculosis. AX compounds target the QcrB subunit of the cytochrome *bc*_1_ terminal oxidase with a different mode of interaction compared to those of known QcrB inhibitors. This study provides the first multifaceted validation of QcrB inhibition by recombineering-mediated allelic exchange, gene expression profiling, and bioenergetic flux studies. It also provides further evidence for the compensatory role of cytochrome *bd* oxidase upon QcrB inhibition. In the absence of cytochrome *bd* oxidase, AX compounds are bactericidal, an encouraging property for future antimycobacterial drug development.

## INTRODUCTION

Mycobacterium tuberculosis, the causative agent of tuberculosis (TB), is at the origin of a severe global health problem. TB is the leading cause of death due to an infectious agent, with an estimated death toll of 1.6 million in 2016 (1). Drug-susceptible TB is typically treated over the course of 6 months with a combination of the first-line drugs isoniazid (INH), rifampin (RIF), pyrazinamide, and ethambutol; however, this regimen has been rendered ineffective nowadays in many cases due to the emergence of drug-resistant TB (1). Treatment of multidrug-resistant (MDR) TB, whose defining characteristics are resistance to both rifampin and isoniazid, requires a regimen of second-line drugs, including a fluoroquinolone and injectable agents, but treatment success rates are lower than 50% (1–3). In addition to resistance to rifampin and isoniazid, extensively drug-resistant (XDR) TB strains are resistant to at least one of the fluoroquinolones and an injectable. XDR TB cure rates are low at 30% (1). Long treatment duration, toxicity issues, drug resistance, and the expense of the second-line regimens are important factors driving the search for new anti-TB drugs with novel mechanisms of action.

To generate new anti-TB candidates, drug screening efforts have employed phenotypic or whole-cell screening approaches recently, whereby compound libraries of new chemical entities are screened against M. tuberculosis for inhibition of bacterial growth in various settings that, ideally, should recapitulate pathophysiological conditions (4–6). Attempts are subsequently made to identify and validate the target(s) of hits to enable and facilitate further compound optimization. The diarylquinoline bedaquiline (BDQ), which targets subunit *c* of mycobacterial ATP synthase (7, 8), emerged from such a compound-to-target approach, has been hailed as a milestone for TB drug discovery, and with its approval in 2012, became the first anti-TB drug to be approved in more than 40 years. The imidazopyrimidine amide Q203 and lansoprazole sulfide (LPZS) both target the QcrB subunit of cytochrome *bc*_1_ oxidase and were identified from intracellular screens with M. tuberculosis-infected macrophages and fibroblasts, respectively (9, 10). The approval of BDQ for the treatment of MDR TB, inclusion of BDQ as a component of the highly promising NIX-TB combination trial for XDR TB treatment (11), and the fact that Q203 is currently in phase I clinical development (2, 12) altogether validate mycobacterial respiration as a new, relevant, and attractive target for an obligate aerobic pathogen.

**FIG 1:**
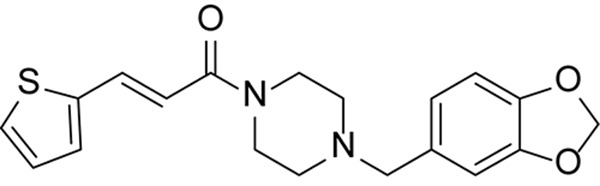
Molecular structure of AX-35 (GW861072X).

In 2013, GlaxoSmithKline published the results of a large phenotypic screening campaign against Mycobacterium bovis BCG and M. tuberculosis, which yielded a total of 177 low-molecular-weight hits (13). One of these compounds was GW861072X (referred to here as AX-35) (Fig. 1), which exhibited potent activity against both M. bovis BCG and M. tuberculosis H37Rv (MIC of 0.3 µM against both species).

Due to its structural simplicity, we considered AX-35 as an attractive starting point for lead optimization and prepared a series of analogs for structure-activity relationship (SAR) studies, the full details of which will be published in a forthcoming report. In this article, we describe the characterization of AX-35 and four of the most potent analogs that have emerged from our SAR work (AX-36 to AX-39) and are active against M. tuberculosis replicating *in vitro* and in infected macrophages. Subsequent work validated the target of these compounds as QcrB, the *b* subunit of the cytochrome *bc*_1_ oxidase, based on the isolation of resistant mutants, cross-resistance studies, transcriptome sequencing (RNA-seq), and bioenergetic flux assays. Finally, further characterization of three AX compounds in an acute mouse model of TB demonstrated *in vivo* efficacy against M. tuberculosis.

## RESULTS

### Initial characterization of AX-35 and its analogs.

The synthesis of AX-35 to AX-39 is described in the supplemental material (for structures, see Table 1). Analog AX-36 served to assess the importance of the position of the sulfur atom in the thiophene ring for antimycobacterial activity, while thiazole derivatives AX-37 and AX-39 were designed to probe the effect of enhanced compound polarity. In addition, replacement of the thiophene ring by a thiazole moiety could mitigate the toxicity risk often associated with thiophene derivatives. For the same reason, the thiophene moiety in AX-35 was also replaced by a phenyl ring, as an established bioisostere (AX-38). As shown in the data in Table 1, AX-35 is the most active compound within this series, with a MIC of 0.05 µg/ml against M. tuberculosis H37Rv; AX-36, AX-37, AX-38, and AX-39 have somewhat higher MIC values ranging from 0.1 to 0.3 µg/ml (Table 1). The activity of AX compounds is selective for pathogenic mycobacteria in the panel of bacterial strains and fungi tested (Table 2). Members of the M. tuberculosis complex were especially susceptible, notably Mycobacterium canettii, followed by Mycobacterium ulcerans. Most of the microorganisms tested were resistant to both AX-35 and AX-36. Mild to no cytotoxicity was observed in human HepG2 cells, resulting in selectivity indices (HepG2 median toxic dose [TD_50_]/MIC) above 250 for all compounds, with AX-35 being the most selective (Table 1). The IC_50_s for M. tuberculosis H37Rv-infected THP-1 macrophages ranged from 0.1 to 1.8 µg/ml (Table 1), reflecting the potent *ex vivo* activity of these molecules. The metabolic stability of the compounds in mouse and human liver microsomes was moderate to low (see Table S1 in the supplemental material).

**TABLE 1:**
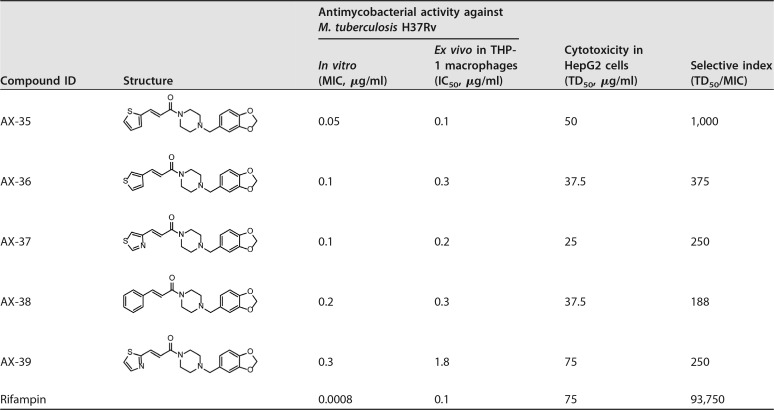
Characterization of AX series: activity against *M. tuberculosis* H37Rv, cytotoxicity, and selective index

**TABLE 2:** Activities of AX-35 and AX-36 against selected microorganisms

Microorganism	MIC (µg/ml) of:		
	AX-35	AX-36	RIF
Bacillus subtilis	>100	>100	0.3
Candida albicans	>100	>100	1.5
Corynebacterium diphtheriae	>100	>100	0.0004
Corynebacterium glutamicum	>100	>100	0.004
Enterococcus faecalis	>100	>100	0.6
Escherichia coli	74	100	6.7
Listeria monocytogenes	>100	>100	0.8
Micrococcus luteus	>100	>100	0.7
Mycobacterium avium	8.2	12.8	25
Mycobacterium bovis BCG	0.8	0.8	0.0008
Mycobacterium canettii STB-L	0.003	0.01	0.002
Mycobacterium marinum	11.5	19.6	0.5
Mycobacterium massiliense	7.7	10.4	26.9
Mycobacterium smegmatis	100	63	1.7
Mycobacterium tuberculosis	0.05	0.1	0.001
Mycobacterium ulcerans	1.6	1.6	0.01
Mycobacterium vaccae	12.1	26.9	2.5
Pseudomonas aeruginosa	>100	>100	1
Pseudomonas putida	>100	>100	0.2
Salmonella enterica serovar Typhimurium	>100	>100	0.7
Staphylococcus aureus	>100	>100	3.8

10.1128/mBio.01276-18.6 TABLE S1 Intrinsic clearance (Cl_int_) and clearance category of AX derivatives in mouse and human liver microsomes. Download Table S1, DOCX file, 0.01 MB. Copyright © 2018 Foo et al. 2018 Foo et al. This content is distributed under the terms of the Creative Commons Attribution 4.0 International license.

### Evidence of AX compounds targeting QcrB of M. tuberculosis.

**FIG 2:**
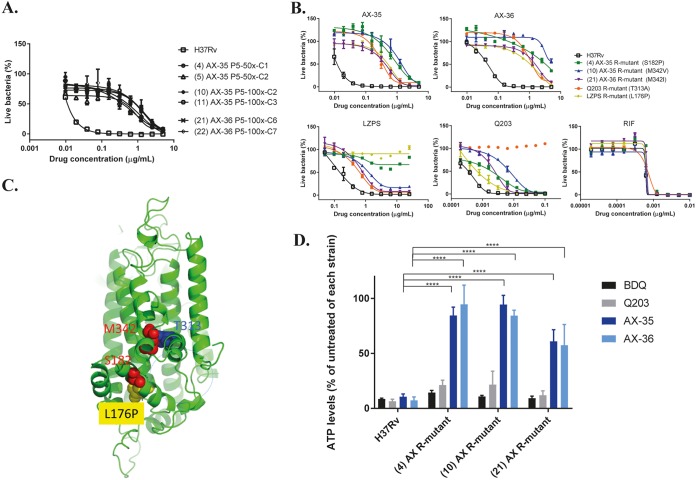
Evidence of AX compounds targeting QcrB of M. tuberculosis. (A) Dose-response curves of isolated mutants resistant to either AX-35 or AX-36 compared to wild-type H37Rv. Three independent 7H9 liquid cultures of M. tuberculosis containing 50× or 100× MIC of AX-35 or AX-36 at passage 5 were plated on 7H10 medium to obtain single colonies. WGS results from AX-resistant mutants reveal mutations in QcrB. (B) Dose-response curves of AX-resistant, Q203-resistant, and LPZS-resistant mutants to AX-35, AX-36, LPZS, Q203, and RIF for cross-resistance studies. The data plotted are presented as mean ± standard deviation (SD); curves are representative of at least two independent experiments. (C) The QcrB protein of M. tuberculosis was modeled on chain A of the crystal structure of the mutant Rhodobacter sphaeroides cytochrome *bc*_1_ oxidase (PDB code 2QJK). Clustering of mutations associated with resistance to AX, Q203, and LPZS occurred around the quinol oxidation site of QcrB, indicated by the dotted blue line. (D) Intracellular ATP levels in H37Rv and AX-resistant strains were measured in the absence and presence of BDQ, Q203, AX-35, and AX-36 at 2.5× MIC after 24 h using BacTiter Glo (Promega). Data from two independent experiments are presented as mean ± SD. Statistical analysis was performed using two-way analysis of variance *(*ANOVA) with Tukey’s multiple-comparison test (****, *P*  < 0.0001).

Initial attempts to raise mutants in M. tuberculosis H37Rv spontaneously resistant to AX-35 and AX-36 on solid 7H10 medium failed, even at exposures of up to 100× MIC. An alternative method of continual passaging of H37Rv in complete 7H9 liquid medium containing AX-35 or AX-36 at concentrations above the MIC, beginning at 2× MIC and increasing to 100× MIC over five passages, proved to be more successful. Increasing MICs reflected the gradual selection of a resistant subpopulation in the culture on constant exposure to the compounds, especially after the fourth and fifth passages. Single clones isolated on 7H10 plates at passage 5 were subsequently tested for resistance to AX-35 or AX-36 by MIC determinations using the resazurin microtiter assay plate (REMA) method (Fig. 2A).

To identify mutations associated with resistance to AX-35 or AX-36, whole-genome sequencing (WGS) analysis was performed on six clones with a 50-fold increase in MIC compared to that of the parental strain H37Rv (Fig. 2A). Three missense mutations in *qcrB* were revealed, leading to nonsynonymous substitutions in QcrB, the *b* subunit of cytochrome *bc*_1_ oxidase. S182P and M342V mutations were associated with AX-35 resistance and M342I with AX-36 resistance. The specific contribution of these substitutions to AX-35 and AX-36 resistance was confirmed by the generation of the same missense mutations in chromosomal *qcrB* of wild-type H37Rv by recombineering and MIC determination (see Fig. S1 in the supplemental material).

10.1128/mBio.01276-18.3 FIG S1 Recombineering of mutations associated with AX resistance in chromosomal M. tuberculosis H37Rv *qcrB*. (A) Two recombinant clones with a strong resistance phenotype to AX compared to wild-type H37Rv or H37Rv containing cotransformed vector pYUB412. (B) Sanger sequencing across *qcrB* confirms insertion of desired base changes in recombinant clones resulting in missense mutations S182P and M342V. The absence of other mutations was confirmed by WGS. Download FIG S1, TIF file, 1.09 MB. Copyright © 2018 Foo et al. 2018 Foo et al. This content is distributed under the terms of the Creative Commons Attribution 4.0 International license.

Since mutations in *qcrB* have also been identified in mutants of M. tuberculosis H37Rv resistant to Q203 and LPZS, two compounds known to target QcrB, cross-resistance studies were performed to investigate if AX-35 and AX-36 behaved in a similar manner. Q203- and LPZS-resistant strains, harboring single-nucleotide polymorphisms (SNPs) leading to T313A and L176P in QcrB, respectively, are cross-resistant to AX-35 and AX-36 (Fig. 2B). These results indicate that the binding mode of the AX compound with QcrB shares some similarities with Q203 and LPZS. However, not all AX-resistant mutants were fully resistant to Q203 and LPZS (Fig. 2B), thus, suggesting a distinct mode of binding for these QcrB inhibitors. On inspection of a model of M. tuberculosis QcrB, residues S182, M342, T313, and L176 were found to cluster around the quinol oxidation site of the enzyme (Fig. 2C), implying that AX-35 and AX-36 bind to QcrB at this pocket, similar to Q203 and LPZS.

As QcrB is one of the respiratory subunits of the cytochrome *bc*_1_ oxidase, a consequence of its inhibition is depletion of ATP levels in the bacterium (9, 10). The intracellular ATP level was measured in wild-type H37Rv after 24 h of exposure to AX-35 or AX-36 and was found to be ∼90% lower than that for the untreated control, similar to the effect of treatment with the ATP synthase inhibitor BDQ or with Q203 under the same conditions (Fig. 2D), thus, indicating that AX compounds do indeed affect ATP levels. ATP was also depleted in BDQ- or Q203-exposed AX-resistant mutants, with ATP levels similar to those in BDQ- or Q203-exposed wild-type M. tuberculosis. However, ATP levels were essentially unaffected in the AX-resistant strains in the presence of AX-35 or AX-36 (Fig. 2D). This demonstrates that the mutations in QcrB associated with AX resistance do not greatly impact the activity of BDQ or Q203, indicating that AX compounds do not have off-target effects on the ATP synthase and that they interact in a different manner with QcrB compared to Q203. Taken together, these findings indicate that QcrB is the direct target of the AX compounds.

### Transcriptional response of M. tuberculosis to AX-35 treatment.

**FIG 3:**
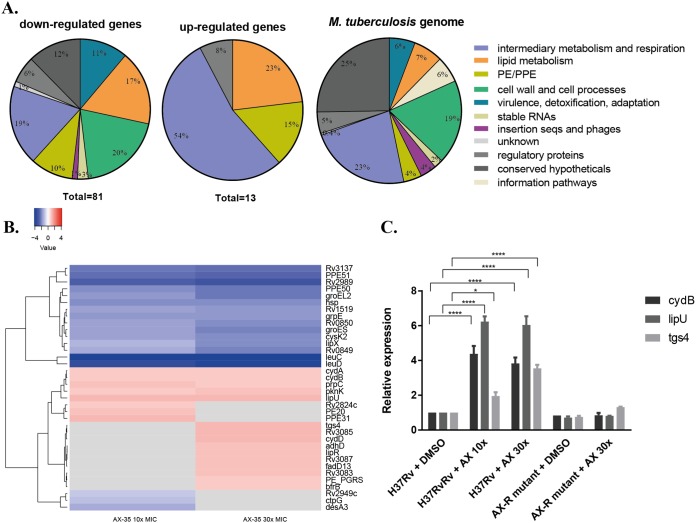
Transcriptional response of M. tuberculosis to AX-35 treatment by RNA-seq. (A) Global transcriptomic response and involvement of different metabolic responses, based on TubercuList classification (https://mycobrowser.epfl.ch/), after exposure of two independent cultures of M. tuberculosis H37Rv to AX-35 at 10× and 30× MIC for 4 h. (B) Heat map representing top significantly differentially regulated M. tuberculosis genes (*P*_adj_ ≤ 0.05). The color scale indicates differential regulation as log_2_ fold change of H37Rv with AX-35 treatment relative to H37Rv with vehicle control, DMSO. Upregulation is indicated in red, downregulation is in blue, and insignificant log_2_ fold change values for the condition are in gray. Data are from two independent experiments. (C) qPCR validation of genes *cydB*, *lipU*, and *tgs4* in H37Rv and AX-resistant mutant strains treated with vehicle (DMSO) or AX-35. Data are presented as mean ± SD from two independent cultures. Statistical analysis was performed using two-way ANOVA with Tukey’s multiple-comparison test (*, *P*  < 0.05; ****, *P*  < 0.0001).

To gain insight into the initial adaptive response of M. tuberculosis to AX-35 treatment, the transcriptomes of wild-type H37Rv exposed to AX-35 at 10× and 30× MIC for a duration of 4 h were examined. Of 81 genes significantly downregulated (fold change [FC] of ≤−2, Benjamini and Hochberg’s adjusted *P* value [*P*_adj_] of ≤0.05), *leuC* and *leuD* (involved in leucine biosynthesis) were the most extensively downregulated (Fig. 3A and B). Thirteen genes were significantly upregulated (FC of ≥2; *P*_adj_ ≤ 0.05) in the presence of AX-35, seven of which were involved in intermediary metabolism and respiration (Fig. 3A and B). The most highly upregulated gene was *lipU*, a gene implicated in lipid hydrolysis. Notably, two main operons in the M. tuberculosis transcriptome were upregulated, namely the *cyd* and *mymA* operons. The *cyd* operon consists of *cydA*, *cydB*, and *cydD* (*cydA* and *cydB* were close to the cutoff with FCs of 1.97 and 1.99, respectively), with *cydA* and *cydB* coding for subunits I and II, respectively, of the cytochrome *bd* oxidase, while *cydDC* encodes an ABC transporter involved in cytochrome *bd* assembly. The *mymA* operon from *rv3083* to *rv3089* (14) includes *tgs4* and *rv3088* encoding triacylglycerol (TAG) synthases (15), *lipR*, *rv3085*, and *rv3086* encoding short-chain dehydrogenases, and an acyl coenzyme A (acyl-CoA) synthase gene, *fadD13*.

To validate the RNA-seq data, real-time quantitative PCR (qPCR) was performed targeting *lipU*, *cydB*, and *tgs4* in both wild-type H37Rv and its AX-resistant mutants after exposure to AX-35 at 30× MIC (Fig. 3C). The upregulation of all three genes in wild-type H37Rv upon AX-35 treatment detected by qRT-PCR is consistent with the RNA-seq data, with *lipU* having the highest relative expression of around 6-fold compared to the control (i.e., H37Rv treated with dimethyl sulfoxide [DMSO] alone). No significant differences in the expression levels of these three genes were measured for the AX-resistant mutant when treated with DMSO or AX-35, therefore establishing that their upregulation and, by extension, the deregulation of genes observed by RNA-seq are indeed a consequence of AX-35 treatment in M. tuberculosis.

### Respiratory response of M. tuberculosis to AX-35 treatment.

**FIG 4:**
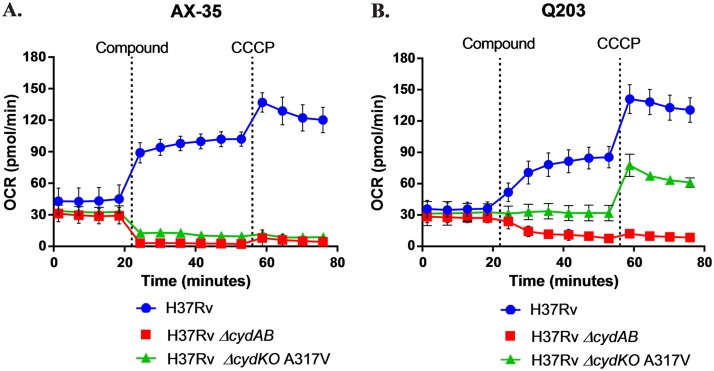
Bioenergetic flux profiles of M. tuberculosis with AX-35 or Q203 treatment. The oxygen consumption rates of wild-type H37Rv, H37Rv Δ*cydAB*, and H37Rv Δ*cyd* KO with a QcrB(A317V) mutation (Q203 resistance SNP) were measured at basal levels, then in the presence of (A) AX-35 (final concentration of 14 µM [100× MIC_50_]) or (B) Q203 (final concentration of 0.3 µM [100× MIC_50_]), and then at maximum capability with the depolarization of the bacterial membrane by the protonophore carbonyl cyanide *m*-chlorophenyl hydrazine (CCCP).

Bioenergetic flux studies were carried out to assess the respiratory response of M. tuberculosis to AX-35, with Q203 used as a control. The oxygen consumption rate (OCR) was first measured in M. tuberculosis H37Rv in the presence of glucose to determine the basal OCR, and the subsequent addition of AX-35 or Q203 resulted in an increase in the OCR (Fig. 4A and B). Finally, the protonophore carbonyl cyanide *m*-chlorophenyl hydrazine (CCCP) was added, depolarizing the bacterial membrane and enabling the measurement of the maximum respiration capability of the bacteria.

The increase in OCR upon the addition of AX-35 or Q203 can be attributed to cytochrome *bd* oxidase, since this increase was no longer observed in the presence of either compound in the Δ*cydAB* strain (Fig. 4A and B). This switch in M. tuberculosis respiration to the *bd* oxidase has previously been validated as a respiratory signature of cytochrome *bc*_1_ oxidase inhibitors such as Q203 (16), and our results indicate a bioenergetic profile of AX-35 similar to that of Q203.

Interestingly, a difference can be observed in the bioenergetic profiles on exposure of the cytochrome *bd* oxidase knockout (KO) strain harboring a QcrB(A317V) mutation to these two QcrB inhibitors. Addition of AX-35 decreases the OCR of this strain, generating a profile similar to that of H37Rv Δ*cydAB* (Fig. 4A), implying that respiration is via the *bd* oxidase branch and that QcrB remains fully inhibited despite the A317V mutation. The basal OCR remains unaffected by the addition of Q203, however (Fig. 4B), which would suggest a partial inhibition of QcrB, and thus the inability of the drug to fully inhibit QcrB due to the mutation. This therefore reveals additional differences in the binding modes of AX-35 and Q203 to QcrB.

Taken together, these results demonstrate the capability of respiratory adaptation of M. tuberculosis via the *bd* oxidase upon treatment with AX-35, generating a respiratory signature similar to that of another established QcrB inhibitor, Q203, albeit highlighting different modes of interaction of the two compounds within the binding pocket.

### Cidality, drug interactions, and *in vivo* efficacy of AX compounds.

The QcrB inhibitors described to date are bacteriostatic due to the compensatory role of the cytochrome *bd* oxidase. To determine the mode of action of AX compounds against M. tuberculosis, minimum bactericidal concentrations (MBCs) were determined in the M. tuberculosis H37Rv and H37Rv Δ*cydAB* strains and the complemented H37Rv Δ*cydAB*::*cydAB* strain (see Fig. S2 in the supplemental material). In H37Rv, AX-35 is bacteriostatic, with an MBC up to a concentration 32-fold higher than the MIC. Conversely, AX-35 was bactericidal against the H37Rv Δ*cydAB* strain at a concentration 4-fold in excess of its MIC. Cidality of AX-35 can be specifically attributed to the lack of the cytochrome *bd* oxidase, as evident from the survival of the bacteria in the complemented strain treated with AX-35.

10.1128/mBio.01276-18.4 FIG S2 Mode of action of AX-35. MBCs (representing 99.9% killed bacteria compared to day 0 [D0]) of AX-35 were measured in the M. tuberculosis H37Rv and H37Rv Δ*cydAB* strains and the H37Rv Δ*cydAB*::*cydAB* complemented strain by exposing mycobacteria to various concentrations of AX-35 (fold MICs respective to each strain) over a duration of 7 days in liquid 7H9 complete medium followed by plating on 7H10 solid medium. CFUs were counted after 4 weeks of incubation at 37°C. cmpd, compound. Download FIG S2, TIF file, 1.05 MB. Copyright © 2018 Foo et al. 2018 Foo et al. This content is distributed under the terms of the Creative Commons Attribution 4.0 International license.

Since synergistic interactions have been previously reported between the cell wall inhibitor PBTZ169 and BDQ (17), as well as between compounds targeting the mycobacterial respiratory chain (16, 18), checkerboard assays for two-drug combinations were performed to determine the nature of interactions between AX-35 with PBTZ169, BDQ, or clofazimine (CFM) in M. tuberculosis H37Rv. The Σ fractional inhibitory concentration (ΣFIC) indices obtained for all three combinations range from 0.8 to 1.6 for concentrations of AX-35 of ≤0.5-fold MIC (see Table S2 in the supplemental material), indicating nonantagonistic, additive interactions.

10.1128/mBio.01276-18.7 TABLE S2 Interactions of AX-35 with PBTZ169, BDQ, or clofazimine (CFM) in M. tuberculosis H37Rv. The data obtained are mean ΣFIC indices ± SD from two independent experiments. Download Table S2, DOCX file, 0.01 MB. Copyright © 2018 Foo et al. 2018 Foo et al. This content is distributed under the terms of the Creative Commons Attribution 4.0 International license.

**FIG 5:**
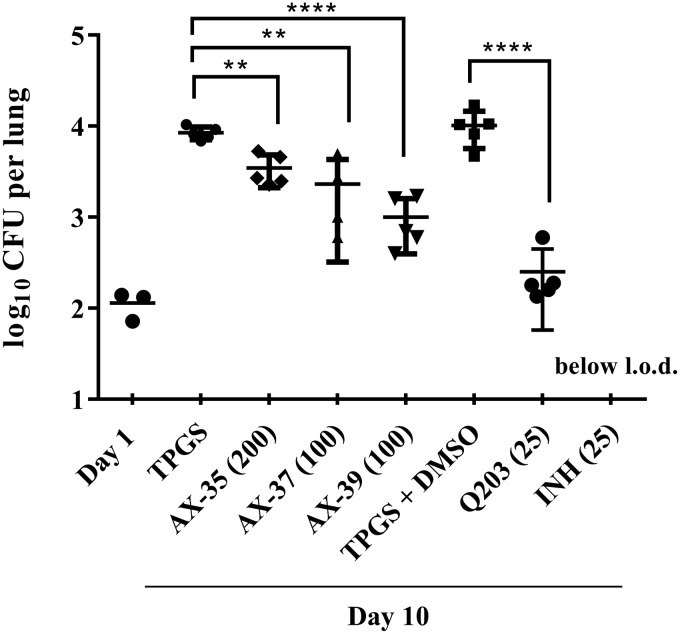
Activity of AX compounds assessed in a mouse model of acute TB. One day after low-dose aerosol M. tuberculosis infection, groups of five mice were treated with vehicle controls (20% TPGS or 20% TPGS with 1% DMSO) or with compounds administered by oral gavage for 10 days daily at the doses indicated (mg/kg). AX compounds were prepared in 20% TPGS, Q203 in 20% TPGS with 1% DMSO, and INH in ddH_2_O. Bacterial burden (CFU) was determined from lung homogenates. Data from one experiment are presented as mean ± SD. Statistical analysis was performed using one-way ANOVA, Tukey’s multiple-comparison test (**, *P*  < 0.01; ****, *P*  < 0.0001). l.o.d., limit of detection.

The activity of AX compounds *in vivo* was determined in mouse models of chronic and acute TB. None of the AX compounds reduced the bacterial burden in mice at the dose of 100 mg/kg of body weight in the chronic model (see Fig. S3 in the supplemental material). In the acute model, however, AX-35 at an oral dose of 200 mg/kg and AX-37 and AX-39 at 100 mg/kg significantly reduced the bacterial loads in mouse lungs by 0.4, 0.6, and 0.9 log_10_, respectively, compared to the d-α-Tocopherol polyethylene glycol 1000 succinate (TPGS) vehicle control (Fig. 5).

10.1128/mBio.01276-18.5 FIG S3 Activity of AX compounds in a chronic mouse model of TB. Bacterial burden (CFU) was determined in the lungs (black columns) and spleens (gray columns) of five mice treated with vehicle control (20% TPGS) or treated with the compounds at the doses as indicated in parentheses (mg/kg) by oral gavage. Day 0 indicates the start of treatment, whereas the rest of the data were obtained from day 28 when treatment ended. Data from one experiment are presented as mean ± SD. Statistical analysis was performed using one-way ANOVA with Dunnett’s multiple-comparison test compared to the nontreated vehicle condition (*, *P*  < 0.05; ***, *P*  < 0.001). Download FIG S3, TIF file, 1.13 MB. Copyright © 2018 Foo et al. 2018 Foo et al. This content is distributed under the terms of the Creative Commons Attribution 4.0 International license.

## DISCUSSION

The compounds investigated in this study are chemically distinct and different from the diverse QcrB inhibitors described in recent years (9, 19–25). Of the three mutations in QcrB identified here in spontaneous mutants resistant to AX-35 and AX-36, namely S182P, M342V, and M342I, the first two have been previously reported (20). Cross-resistance studies and bioenergetic flux assays highlight influential residues for the interaction of AX-35 and AX-36 with QcrB, which occurs in the same binding site as Q203 and LPZS. In particular, mutations S182P, M342V/I, T313A, and L176P impair the interaction of AX-35 and AX-36 with QcrB, resulting in resistance of M. tuberculosis to the compounds. The substitution A317V, however, does not interfere with this interaction. This highlights the different modes of interaction of AX-35 and AX-36 with QcrB compared with Q203 or LPZS, and this information will be useful for further optimization of the compounds through structure-based drug design.

In addition to cross-resistance studies, transcriptomic and bioenergetic flux studies provided further evidence of QcrB as the primary target of AX-35/36. Treatment of M. tuberculosis with AX-35 results in the upregulation of the *cydABD* operon, but not the *dosR* regulon, which is consistent with the transcriptomic signature of respiratory inhibitors specifically inhibiting the cytochrome *bc*_1_-*aa*_3_ terminal oxidase (26). In line with this, exposure of M. tuberculosis to AX-35 results in an increase in OCR due to activity of the cytochrome *bd* oxidase, generating a bioenergetic profile similar to that of the respiratory signature of cytochrome *bc*_1_ oxidase inhibitors such as Q203 (16).

Cytochrome *bd* oxidase is non-proton translocating and, thus, the less bioenergetically efficient of the two terminal oxidases of M. tuberculosis (27). It has been demonstrated that the *bd* oxidase is upregulated under conditions in which the function of the cytochrome *bc*_1_-*aa*_3_ is compromised (20, 28–30). In addition, inhibitors of respiration such as BDQ and QcrB inhibitors have markedly improved activity upon deletion or inhibition of *bd* oxidase (20, 31–33). The pronounced compensatory role of this alternate oxidase in the respiratory adaptation of M. tuberculosis for its survival is also apparent in our study, where the absence of *bd* oxidase is associated with AX-mediated bactericidal activity. Targeting both terminal oxidases simultaneously may therefore be a novel and effective strategy against M. tuberculosis. Although the increase in respiration, upregulation of cytochrome *bd* oxidase, and enhanced killing of Δ*cydAB* mutants upon inhibition of ATP synthase by BDQ in Mycobacterium smegmatis (34) have been attributed to the ionophoric/off-target effect of BDQ (35), it is unlikely that AX compounds behave in a similar manner to uncouplers, based on the bioenergetic flux data.

It is also of note that AX treatment of M. tuberculosis results in the upregulation of *tgs* genes (*tgs4* and *rv3087*) and lipases (*lipU* and *lipR*). *tgs* genes are involved in the biosynthesis of triacylglycerol (TAG), the predominant energy source for M. tuberculosis in host macrophages (15, 36), while *lip* genes encode lipolytic enzymes thought to be involved in the utilization of host TAG (37). Such upregulation of *tgs* and *lip* genes has been observed in various models of host stress (38, 39), and similarly AX-induced stress remodels central carbon metabolism in M. tuberculosis toward lipid metabolism.

The compounds investigated here show promising properties as anti-TB agents. AX-35 to AX-39 demonstrated potent activity against M. tuberculosis *in vitro* and in macrophages, while having mild to no cytotoxicity. When tested in an acute mouse model of TB, AX-35, AX-37, and AX-39 were capable of reducing the bacterial burden in the lungs of mice. This *in vivo* activity can likely be improved further by addressing metabolic stability issues, such as those observed in mouse microsomes, although the compounds appear to be more stable in human microsomes.

The fact that activity of the AX compounds was only observed in the acute rather than the chronic mouse model of TB could be attributed to the difference in expression levels of the terminal oxidases found between these two stages of M. tuberculosis lung infection (29). The cytochrome *bc*_1_-*aa*_3_ oxidase is more highly expressed than the *bd* oxidase during the exponential phase of growth of M. tuberculosis in mice, whereas it is downregulated as a response to host immunity and remains downregulated as the bacteria enter a nonreplicating state in the chronic phase (29). Thus, targeting QcrB during the exponential phase of M. tuberculosis growth would have a bigger impact on bacterial viability despite any compensatory response by *bd* oxidase. Another potential application of the AX inhibitors could be in the treatment of the human diseases Buruli ulcer and leprosy as their causative agents, M. ulcerans and Mycobacterium leprae, respectively, lack the cytochrome *bd* oxidase.

In conclusion, our SAR data prove that structural changes to AX-35 (GW861072X) can lead to improved *in vivo* activity against M. tuberculosis and that the arylvinylpiperazine amide series target the QcrB subunit of the cytochrome *bc*_1_-*aa*_3_ oxidase. As part of the mycobacterial respiratory chain, QcrB is an attractive target for combination therapy. Furthermore, the biochemical information obtained from this study could guide future lead optimization work to enhance potency and stability of the compounds.

## MATERIALS AND METHODS

### Drugs used in this study.

Q203 was synthesized as described before (9, 40), LPZS was purchased from Santa Cruz Biotechnology, Toronto Research Chemicals, Inc., BDQ was a gift from Janssen Pharmaceutica NV, and PBTZ169 (macozinone) was provided by Innovative Medicines for Tuberculosis, while CFM, INH, and RIF were from Sigma-Aldrich.

### Culture conditions of M. tuberculosis strains, other bacteria, and eukaryotic cell lines.

Mycobacterial strains were grown at 37°C in Middlebrook 7H9 broth (Difco) supplemented with 0.2% glycerol, 0.05% Tween 80, and 10% albumin dextrose catalase (ADC) (7H9 complete) or on 7H10 agar plates supplemented with 0.5% glycerol and 10% oleic ADC. Bacillus subtilis, Candida albicans, Corynebacterium diphtheriae, Corynebacterium glutamicum, Escherichia coli, Micrococcus luteus, Pseudomonas putida, Salmonella enterica serovar Typhimurium, and Staphylococcus aureus were grown in LB broth. Enterococcus faecalis, Listeria monocytogenes, and Pseudomonas aeruginosa were grown in brain heart infusion (BHI) broth. HepG2 cells were grown in Dulbecco’s modified Eagle’s medium (DMEM [Gibco]) supplemented with 10% fetal bovine serum at 37°C with 5% CO_2_. THP-1 macrophages were grown in RPMI medium supplemented with 10% fetal bovine serum and 1 mM sodium pyruvate at 37°C with 5% CO_2_.

### Determination of MICs.

MICs were determined using the resazurin reduction microplate assay (REMA) as previously described (41). Strains were grown to log phase (optical density at 600 nm [OD_600_] of 0.4 to 0.8) and diluted to an OD_600_ of 0.0001. Two-fold serial dilutions of each test compound were prepared in 96-well plates containing 100 µl of bacteria per well (3 × 10^3^ cells per well). Plates were incubated at either 30 or 37°C as required with appropriate incubation times (e.g., 6 days for M. tuberculosis at 37°C). Ten microliters of resazurin (0.0025% wt/vol) was added to each well, after which the fluorescence intensity of the resorufin metabolite (excitation/emission, 560/590 nm) was read using an Infinite F200 Tecan plate reader. MIC values representing 90% growth inhibition were determined by a nonlinear fitting of the data to the Gompertz equation using GraphPad Prism.

### Cytotoxicity for HepG2 cells.

Human HepG2 cells (4,000 cells/well) were incubated for 3 days with 2-fold serially diluted compounds at 37°C under an atmosphere of 5% CO_2_. Cell viability was determined by the addition of resazurin (0.0025% wt/vol) for 4 h at 37°C, and the fluorescence intensity was measured as in REMA.

### Assessment of drug activity in THP-1 macrophages.

THP-1 human monocytic cells (10^5^/well) were seeded into 96-well plates and incubated with 100 nM phorbol-12-myristate-13-acetate (PMA) overnight to stimulate macrophage differentiation. Differentiated macrophages were infected with M. tuberculosis H37Rv grown to log phase (OD_600_ of 0.4 to 0.8) at a multiplicity of infection (MOI) of 5. Extracellular bacteria were removed after 3 to 4 h of incubation at 37°C with 5% CO_2_ by removing the RPMI medium and washing with phosphate-buffered saline (PBS). The compounds to be tested were prepared in separate 96-well plates by 2-fold serial dilutions in a final volume of 100 µl RPMI, which was then transferred to the plates of infected THP-1 macrophages. Plates were sealed and incubated for 48 h at 37°C with 5% CO_2_. Ten microliters of PrestoBlue (Thermo Fischer Scientific) was added, and plates were incubated for up to 1 h at 37°C with 5% CO_2_ before the fluorescence intensity (excitation/emission, 560/590 nm) was measured using the Infinite F200 Tecan plate reader. Dose-response curves were plotted, and 50% inhibitory concentrations (IC_50_s) were obtained using a nonlinear regression fit equation (log[inhibitor] versus response, variable slope) in GraphPad Prism.

### Microsomal stability studies.

Metabolic stability of the compounds was measured based on intrinsic clearance (Cl_int_) in mouse and human liver microsomes as previously described (42). The final compound concentration in the mixture of microsomes and NADPH regeneration system was 2 µg/ml, and a mixture without NADPH regeneration was also prepared for each compound as a control of the stability of the compound with time. Carbamazepine and nifedipine at 2 µg/ml were used as low- and high-Cl_int_ controls, respectively.

### Mouse studies.

To assess compound activity in an acute mouse model of TB, female BALB/c mice from Charles River Laboratories (7 to 8 weeks old, 20 g, 5 mice per group) were aerosol infected with M. tuberculosis H37Rv at a low dose and treated by oral gavage the following day with either vehicle controls (20% TPGS or 20% TPGS containing 1% DMSO) or test compounds once daily for 10 days. AX compounds were prepared in 20% TPGS, Q203 in 20% TPGS containing 1% DMSO (as described in reference 32), and INH in double-distilled water (ddH_2_O). Compounds were ground with a pestle and mortar followed by sonication at room temperature for 30 min. Compound solutions were stored at 4°C and prepared freshly after 5 days. The day after the final treatment, mice were sacrificed and serial dilutions of lung homogenates were plated on 7H10 agar containing 10 µg/ml cycloheximide and 50 µg/ml ampicillin. Experiments were approved by the Swiss Cantonal Veterinary Authority (authorization no. 3082).

### Isolation and characterization of AX-resistant mutants.

AX-resistant mutants of M. tuberculosis H37Rv were isolated from 7H9 cultures over 5 passages with increasing concentrations of AX-35 or AX-36 starting from 2×, 5×, and 10× MIC to final concentrations of 50× and 100× MIC. Single colonies were obtained from three independent cultures by streaking on 7H10 agar plates, and resistance to AX was measured by REMA. Genomic DNA extraction was performed using the QiaAMP UCP pathogen minikit (Qiagen) as per the manufacturer’s instructions. Whole-genome sequencing was performed using Illumina technology with sequencing libraries prepared using the KAPA HyperPrep kit (Roche) and sequenced on an Illumina HiSeq 2500 instrument. All raw reads were adapter and quality trimmed with Trimmomatic v0.33 (43) and mapped onto the M. tuberculosis H37Rv reference genome (RefSeq no. NC_000962.3) using Bowtie2 v2.2.5 (44). The bamleftalign program from the FreeBayes package v0.9.20-18 (45) was used to left-align indels. Reads with a mapping quality below 8 and duplicate reads were omitted.

### Variant analysis.

Variant calling was done using VarScan v2.3.9 (46) using the following cutoffs: minimum overall coverage of 10 nonduplicated reads, minimum of 5 nonduplicated reads supporting the SNP, base quality score of >15, and an SNP frequency above 30%. The rather low thresholds, especially the SNP frequency, were deliberately chosen to avoid missing potential variants in regions where alignment was difficult or in case of a mixed population. All putative variants unique to the mutant strains were manually checked by inspecting the alignments.

### Recombineering for target confirmation.

Insertion of mutations associated with AX resistance in chromosomal *qcrB* of M. tuberculosis was done based on a recombineering method (47). The desired mutations were centered in lagging-strand oligonucleotides of 70 nucleotides. M. tuberculosis H37Rv containing plasmid pJV53 was grown to an OD_600_ of 0.8 in 7H9 liquid medium containing 25 µg/ml kanamycin and exposed to 0.2% acetamide for 24 h and 0.2 M glycine for 16 h. Competent cells were prepared and cotransformed with 100 ng of lagging-strand oligonucleotide and 50 ng of pYub412 carrying a hygromycin selection marker. After rescue for 3 days at 37°C, cells were plated on 7H10 plates containing hygromycin at 50 µg/ml, and transformants were evaluated for resistance to AX by REMA. Desired mutations associated with AX resistance were subsequently confirmed by Sanger sequencing across *qcrB*, and the absence of other mutations was confirmed by whole-genome sequencing.

### Modeling of M. tuberculosis QcrB.

The QcrB protein of M. tuberculosis was modeled using the homology modeling web server (SWISS) (48) and chain A of the crystal structure of the mutant Rhodobacter sphaeroides cytochrome *bc*_1_ oxidase (PDB code 2QJK). Illustrations were made using Pymol software (49).

### Quantification of intracellular ATP.

Log-phase cultures of wild-type H37Rv or AX-resistant mutants (about 10^6^ CFU/ml) were exposed to test compounds at 2.5× MIC for 24 h in a final volume of 100 µl and incubated with BacTiter Glo reagent (Promega) (4:1 vol/vol) for 5 min in the dark. Luminescence was measured on a TECAN Infinite M200 in relative light units (RLU) with an integration time of 1 s.

### Total RNA extraction and RNA-seq.

Wild-type and AX-resistant H37Rv cultures were grown to mid-log phase and exposed to DMSO (vehicle control) or compounds for 4 h at 37°C. Cells were harvested by centrifugation and pellets were stored with 1 ml of TRIzol reagent (Thermo Fisher Scientific) at −80°C until further processed. Cells were lysed by bead-beating, and total RNA was extracted by phenol-chloroform with DNase treatment (RQ1 RNase-free DNase; Promega). Library preparation was done using the Ribo-zero rRNA removal kit (Illumina) for Gram-positive bacteria to deplete rRNA from total RNA. Two biological replicates for each strain were prepared for RNA-seq. Reads were adapter and quality trimmed with Trimmomatic v0.33 (43) and mapped onto the M. tuberculosis H37Rv reference genome (RefSeq no. NC_000962.3) using Bowtie2 v2.2.5 (44) Counting of reads over features was done with featureCounts from the Subread package v1.4.6 (50). DESeq2 (51) was used to infer differentially expressed genes.

### qPCR.

cDNA was prepared from total RNA using the SuperScript III first-strand synthesis kit (Invitrogen) and analyzed by qPCR for targeted gene expression in duplicates using Power SYBR Green PR master mix (Applied Biosystems) on a QuantStudio 5 real-time PCR system (Thermo Fisher Scientific). *sigA* was used as a housekeeping gene for normalization, and the threshold cycle (ΔΔ*C_T_*) method was used for quantification.

### Extracellular flux analysis.

All strains of M. tuberculosis used were cultured in Middlebrook 7H9 medium (Difco) supplemented with 10% OADC (oleic acid-albumin-dextrose-catalase [Difco]) and 0.01% tyloxapol (Sigma) at 37°C to an OD_600_ of ∼0.6 to 0.8. M. tuberculosis H37Rv was obtained from BEI Resources (NR-123), and M. tuberculosis H37Rv Δ*cydAB* (52) and H37Rv Δ*cyd* KO A317V (20) were gifts from Digby Warner and Helena Boshoff, respectively. The M. tuberculosis oxygen consumption rate (OCR) was measured using the Seahorse XF96 analyzer (Agilent) as previously described (16). In short, M. tuberculosis bacilli were adhered to the bottom of a XF96 cell culture microplate (Agilent) at a density of 2 × 10^6^ bacilli/well using Cell-Tak cell adhesive (Corning). Extracellular flux analysis was carried out in unbuffered 7H9 medium at pH 7.35 containing 0.2% glucose. Basal OCR was measured for ∼19 min before the automatic addition, through the drug ports of the XF96 sensory cartridge (Agilent), of either Q203 (final concentration of 0.3 µM [100× the MIC_50_]) or AX-35 (final concentration of 14 µM [100× the MIC_50_]) to the three different M. tuberculosis strains. Q203 was a gift from Helena Boshoff. The deviations from basal respiration, caused by compound addition, were measured for ∼35 min before the addition of the uncoupler CCCP (final concentration of 2 µM [Sigma]) to induce maximal OCR, after which OCR was measured for a final ∼19 min. The points of compound addition are indicated by vertical dotted lines on Fig. 4. OCR data points are representative of the average OCR after 3 min of continuous measurement, with the error calculated automatically by the Seahorse Wave Desktop 2.3.0 software (Agilent) from the OCR measurements from at least four replicate wells. OCR plots are representative of two independent experiments performed, and data representation was done using GraphPad Prism 7.02.

### Data availability.

Sequence data have been deposited in the NCBI Sequence Read Archive under accession no. SRP142469 (https://www.ncbi.nlm.nih.gov/sra/SRP142469). RNA-seq data have been deposited in the NCBI Gene Expression Omnibus under accession no. GSE113683 (https://www.ncbi.nlm.nih.gov/geo/query/acc.cgi?acc=GSE113683).

10.1128/mBio.01276-18.1 TEXT S1 Supplemental methods. Assessment of efficacy in a chronic mouse model of TB. Checkerboard assays for two-drug combinations containing AX-35. Download Text S1, DOCX file, 0.1 MB. Copyright © 2018 Foo et al. 2018 Foo et al. This content is distributed under the terms of the Creative Commons Attribution 4.0 International license.

10.1128/mBio.01276-18.2 TEXT S2 Supplemental material. Synthesis and characterization data of AX-35 and analogs: general information on ^1^H-, ^13^C-, and ^31^P-nuclear magnetic resonance (NMR) spectra. Download Text S2, DOCX file, 2.9 MB. Copyright © 2018 Foo et al. 2018 Foo et al. This content is distributed under the terms of the Creative Commons Attribution 4.0 International license.

10.1128/mBio.01276-18.8 TABLE S3 List of primers used in this study. Download Table S3, DOCX file, 0.1 MB. Copyright © 2018 Foo et al. 2018 Foo et al. This content is distributed under the terms of the Creative Commons Attribution 4.0 International license.
