# Supplementary material for: Arylvinylpiperazine Amides, a New Class of Potent Inhibitors Targeting QcrB of Mycobacterium tuberculosis
Source: mBio. 2018 Oct 9;9(5):e01276-18. doi: 10.1128/mBio.01276-18 (PMC6178619; doi:10.1128/mBio.01276-18)
Supplement: TABLE S1 [file mbo005184080st1.docx]

# SUPPLEMENTARY TABLES

**Table S1** Intrinsic clearance (Cl_int_) and clearance category of AX derivatives in mouse and human liver microsomes

|  |  |  |  |  |  |
| --- | --- | --- | --- | --- | --- |
|  | **Mouse microsomes** | |  | **Human microsomes** | |
|  | Clearance Category | Clint (µl/min/mg) |  | Clearance Category | Clint (µl/min/mg) |
| AX-35 | high | 119.6 |  | medium | 21.7 |
| AX-36 | high | 116.8 |  | medium | 16.8 |
| AX-37 | N.D. | N.D. |  | N.D. | N.D. |
| AX-38 | high | 63.8 |  | medium | 13.3 |
| AX-39 | N.D. | N.D. |  | N.D. | N.D. |
| Carbamazepine | low | 0.6 |  | low | 0.4 |
| Nifedipine | high | 104.6 |  | high | 121.1 |

N.D. = not determined
