# Supplementary figures and images for: Arylvinylpiperazine Amides, a New Class of Potent Inhibitors Targeting QcrB of Mycobacterium tuberculosis
Source: mBio. 2018 Oct 9;9(5):e01276-18. doi: 10.1128/mBio.01276-18 (PMC6178619; doi:10.1128/mBio.01276-18)

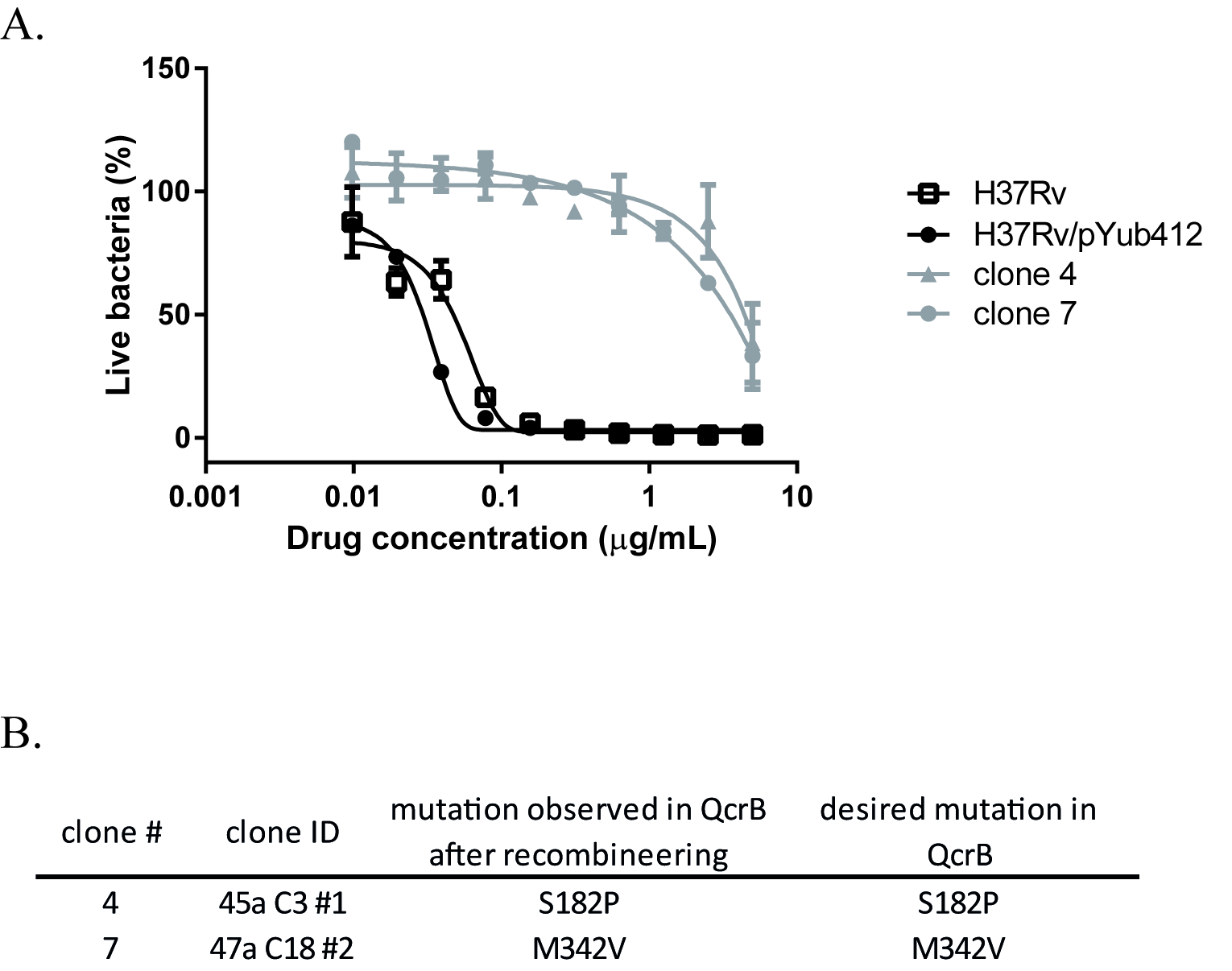

Supplement: FIG S1 [file mbo005184080sf1.tif]

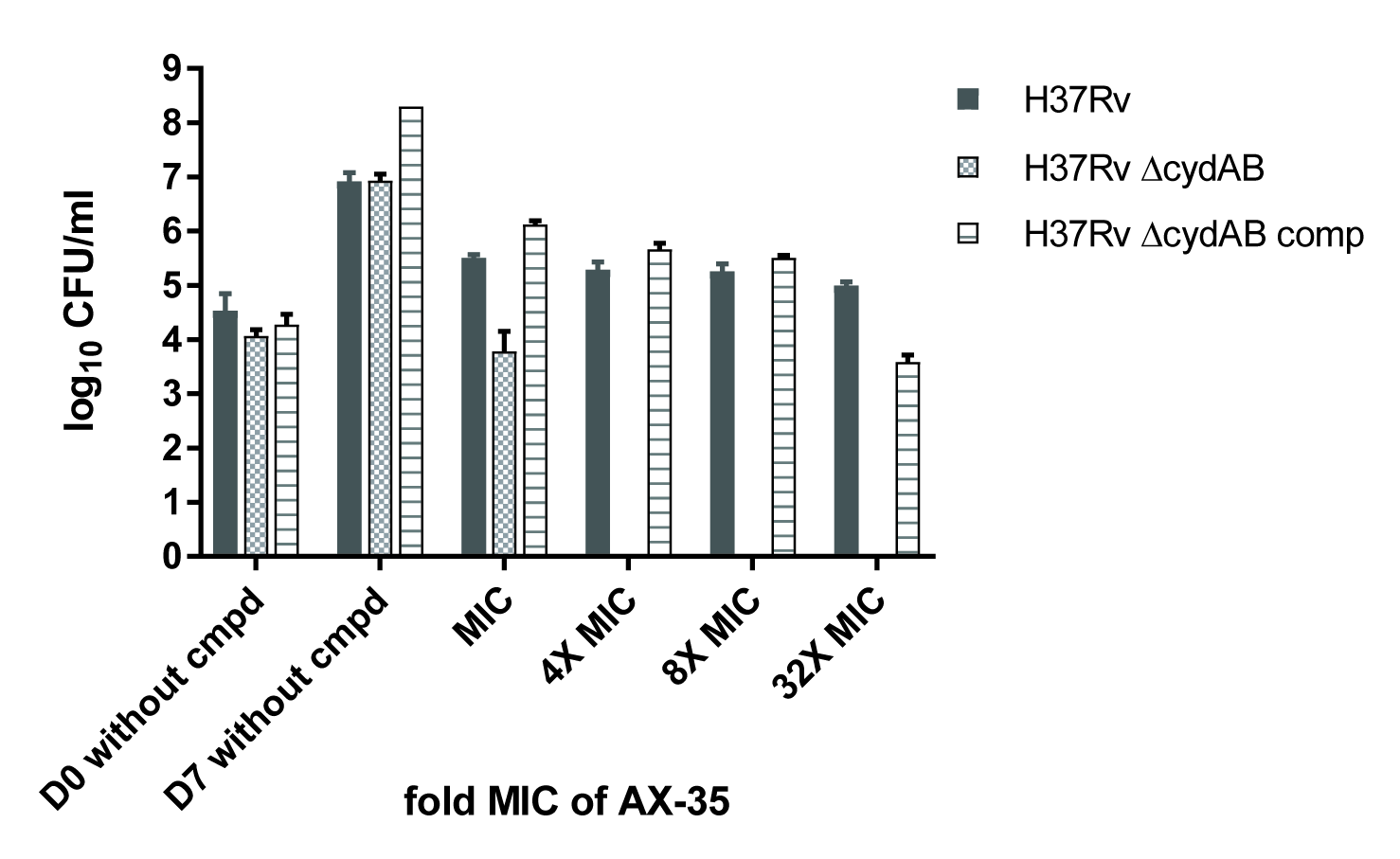

Supplement: FIG S2 [file mbo005184080sf2.tif]

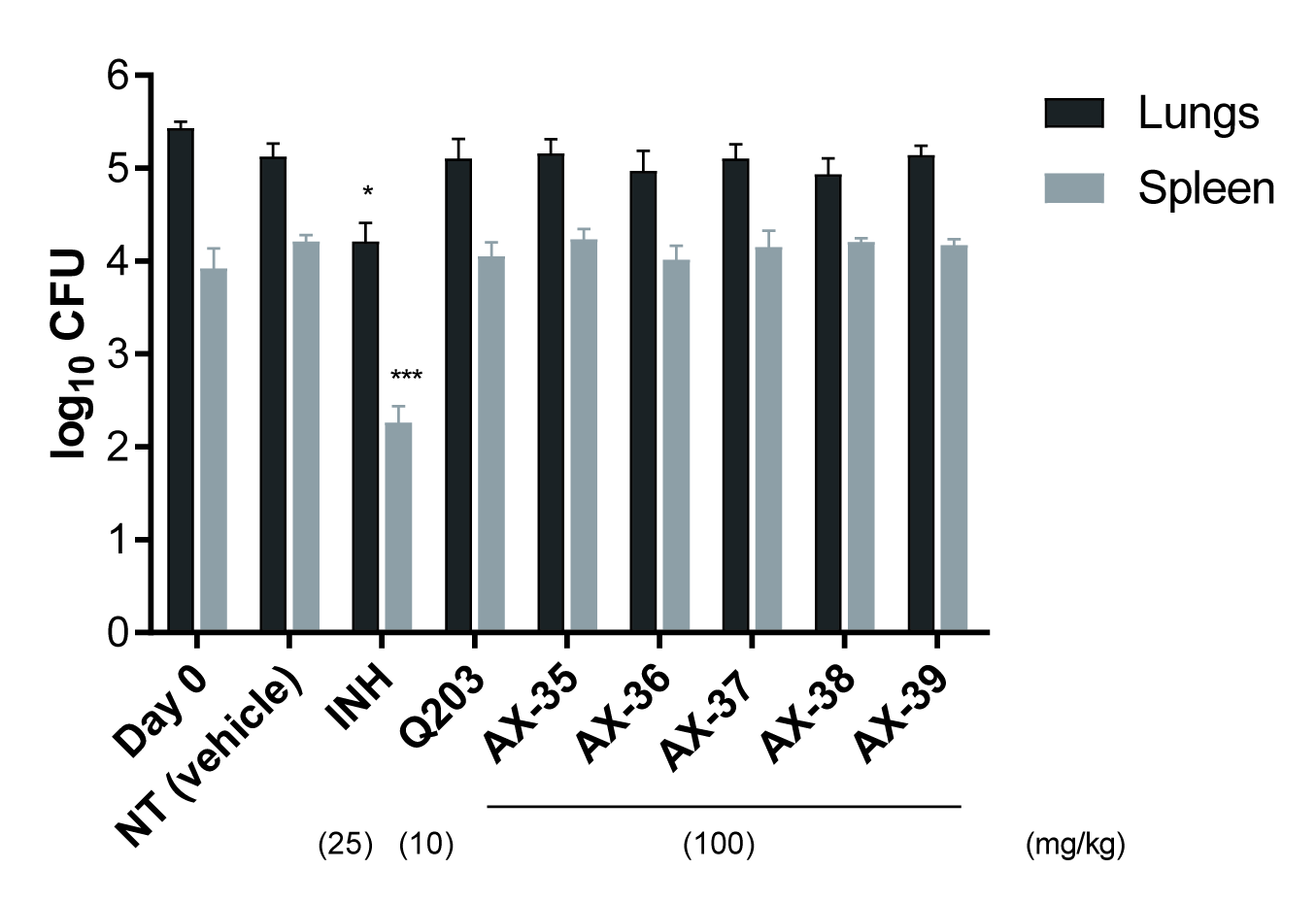

Supplement: FIG S3 [file mbo005184080sf3.tif]
