# Supplementary material for: Arylvinylpiperazine Amides, a New Class of Potent Inhibitors Targeting QcrB of Mycobacterium tuberculosis
Source: mBio. 2018 Oct 9;9(5):e01276-18. doi: 10.1128/mBio.01276-18 (PMC6178619; doi:10.1128/mBio.01276-18)
Supplement: TABLE S2 [file mbo005184080st2.docx]

# SUPPLEMENTARY TABLES

**Table S2** Interactions of AX-35 with PBTZ169, BDQ or CFM in M. tuberculosis H37Rv. Data obtained are mean ΣFIC indices ± SD from two independent experiments.

|  |  |  |  |  |  |  |  |  |
| --- | --- | --- | --- | --- | --- | --- | --- | --- |
| **fold MIC of AX-35** | **AX-35 with PBTZ169** | |  | **AX-35 with BDQ** | |  | **AX-35 with CFM** | |
|  | Mean ΣFIC Index | SD |  | Mean ΣFIC Index | SD |  | Mean ΣFIC Index | SD |
| 0.5 | 1.63 | 0.08 |  | 1.06 | 0.13 |  | 0.79 | 0.07 |
| 0.25 | 1.31 | 0.04 |  | 1.33 | 0.00 |  | 0.80 | 0.15 |
| 0.125 | 1.03 | 0.24 |  | 1.17 | 0.00 |  | 0.92 | 0.29 |
| 0.0625 | 0.83 | 0.28 |  | 1.00 | 0.17 |  | 1.02 | 0.13 |
