# Supplementary material for: Arylvinylpiperazine Amides, a New Class of Potent Inhibitors Targeting QcrB of Mycobacterium tuberculosis
Source: mBio. 2018 Oct 9;9(5):e01276-18. doi: 10.1128/mBio.01276-18 (PMC6178619; doi:10.1128/mBio.01276-18)
Supplement: TEXT S1 [file mbo005184080s1.docx]

# SUPPLEMENTARY METHODS

**Assessment of efficacy in a chronic mouse model of TB.** Female BALB/c mice (5-6 weeks old, 20g) from Charles River Laboratories were infected with a low-dose aerosol of *M. tuberculosis* H37Rv. Treatment was initiated 4 weeks after the infection by oral gavage 5 days a week over 4 weeks. INH was prepared at 25 mg/kg in ddH2O, Q203 at 10 mg/kg in TPGS 20% (as described in (1) and AX derivatives at 10 mg/kg in TPGS 20%. The non-treated group received the vehicle TPGS 20% alone. Compounds were ground with a pestle and mortar followed by sonication at room temperature for 30 min. Compound solutions were stored at 4^o^C and freshly prepared at the start of each week. The day after the final treatment, all groups were sacrificed and serial dilutions of lung and spleen homogenates were plated on 7H10 agar containing 10 µg/ml cyclohexamide and 25 µg/ml ampicillin. Experiments were approved by the Swiss Cantonal Veterinary Authority (authorisation number 3082).

**Checkerboard assays for two-drug combinations containing AX-35.** Interactions between AX-35 and PBTZ169, BDQ or CFM were determined using the checkerboard assay (2, 3). *M. tuberculosis* H37Rv was grown to log-phase in 7H9 media and diluted to an OD_600_ of 0.0001. 75 µl of bacterial suspension (about 10^3^ cells) was added per well of a 96-well plate. PBTZ169, BDQ or CFM were two-fold serially diluted column-wise (1^st^ compound). AX-35 was prepared in 7H9 medium starting at 8x MIC and serial dilutions were made to 0.125x. 25 µl of diluted compound at each concentration was added to a row of the 96-well plate. After incubation of the plates for 6 days at 37oC, 10 µl of 0.025% resazurin was added. The fluorescence intensity was read after 24 h incubation using an Infinite F200 Tecan plate reader. For rows where an MIC value could be determined, the fractional inhibitory concentration index (ΣFIC index) was calculated using the equation ΣFIC index = FIC_1st compound_ + FIC_AX-35_ = (MIC of 1^st^ compound, tested in combination)/(MIC of 1^st^ compound, alone) + (MIC of AX-35, tested in combination)/(MIC of AX-35, alone). ΣFIC index ≤ 0.5 indicates synergism, 0.5 < ΣFIC index ≤ 4 additivity, and ΣFIC index > 4 antagonism.

# REFERENCES

1. Pethe K, Bifani P, Jang J, Kang S, Park S, Ahn S, Jiricek J, Jung J, Jeon HK, Cechetto J, Christophe T, Lee H, Kempf M, Jackson M, Lenaerts AJ, Pham H, Jones V, Seo MJ, Kim YM, Seo M, Seo JJ, Park D, Ko Y, Choi I, Kim R, Kim SY, Lim S, Yim S-A, Nam J, Kang H, Kwon H, Oh C-T, Cho Y, Jang Y, Kim J, Chua A, Tan BH, Nanjundappa MB, Rao SPS, Barnes WS, Wintjens R, Walker JR, Alonso S, Lee S, Kim J, Oh S, Oh T, Nehrbass U, Han S-J, No Z, Lee J, Brodin P, Cho S-N, Nam K, Kim J. 2013. Discovery of Q203, a potent clinical candidate for the treatment of tuberculosis. Nat Med 19:1157.

2. Reddy VM, Einck L, Andries K, Nacy CA. 2010. In Vitro Interactions between New Antitubercular Drug Candidates SQ109 and TMC207. Antimicrob Agents Chemother 54:2840–2846.

3. Lechartier B, Hartkoorn RC, Cole ST. 2012. In Vitro Combination Studies of Benzothiazinone Lead Compound BTZ043 against Mycobacterium tuberculosis. Antimicrob Agents Chemother 56:5790–5793.
