# Supplementary material for: Arylvinylpiperazine Amides, a New Class of Potent Inhibitors Targeting QcrB of Mycobacterium tuberculosis
Source: mBio. 2018 Oct 9;9(5):e01276-18. doi: 10.1128/mBio.01276-18 (PMC6178619; doi:10.1128/mBio.01276-18)
Supplement: TEXT S2 [file mbo005184080s2.docx]

# Supplemental material for

# Arylvinylpiperazine amides, a new class of potent inhibitors targeting QcrB of *Mycobacterium tuberculosis*

Caroline S. Foo^1§^, Andréanne Lupien^1§^, Maryline Kienle^2^, Anthony Vocat^1^, Andrej Benjak^1^, Raphael Sommer^1^, Dirk A. Lamprecht^3#^, Adrie JC Steyn^3,4^, Kevin Pethe^5^, Jérémie Piton^1^, Karl-Heinz Altmann^2^, Stewart T. Cole^1^

^1^Global Health Institute, École Polytechnique Fédérale de Lausanne, Switzerland

^2^Institute of Pharmaceutical Sciences, Department of Chemistry and Applied Biosciences, ETH Zürich, Switzerland

^3^Africa Health Research Institute, South Africa

^4^Department of Microbiology, University of Alabama at Birmingham, Birmingham, AL, United States

^5^ Lee Kong Chian School of Medicine and School of Biological Sciences, Nanyang Technological University, Singapore

^§^These authors contributed equally to the design and execution of experiments

^#^ current affiliation: Janssen Pharmaceutica, Turnhoutweg 30, 2340, Beerse, Belgium

**Corresponding author:** Prof. S. T. Cole, École Polytechnique Fédérale de Lausanne, Global Health Institute, Station 19, CH-1015 Lausanne, Switzerland

[stewart.cole@epfl.ch](mailto:stewart.cole@epfl.ch)

# SYNTHESIS AND CHARACTERIZATION DATA OF AX-35 AND ANALOGS

**General information.** All reactions were performed under an argon atmosphere using flame-dried glassware and standard syringe/septa techniques. CH_2_Cl_2_ and THF used for reactions were distilled under argon prior to use (CH_2_Cl_2_ from CaH_2_ and THF from Na/benzophenone). All other absolute solvents were purchased as anhydrous grade from Acros (puriss.; dried over molecular sieves; H_2_O <0.005 %) and used without further purification. Solvents for extractions, flash column chromatography and thin layer chromatography (TLC) were either purchased as commercial grade and distilled prior to use or purchased as HPLC grade. All other commercially available reagents were used without further purification. Reactions were magnetically stirred and monitored by TLC performed on Merck TLC aluminum sheets (silica gel 60 F254). Spots were visualized with UV light (λ = 254 nm) or through staining with Ce_2_(SO_4_)_3_/phosphomolybdic acid/H_2_SO_4_ (CPS), or KMnO_4_/K_2_CO_3_. NaHCO_3_ and brine (NaCl) refer to aqueous saturated solutions. Chromatographic purification of products was performed using either Sigma-Aldrich or SiliCycle silica gel 60 for preparative column chromatography (particle size 40-63 μm). ^1^H-, ^13^C- and ^31^P-NMR spectra were recorded on a Bruker AV-400 400 MHz. Chemical shifts (δ) are reported in ppm and are referenced to chloroform (δ 7.26 ppm for ^1^H, δ 77.16 ppm for ^13^C). All ^13^C-NMR spectra were measured with complete proton decoupling. Data for NMR spectra are reported as follows: s = singlet, d = doublet, t = triplet, q = quartet, quint = quintet, sept = septet, m = multiplet, br = broad signal, app. = apparent. Infrared spectra (IR) were recorded on a Jasco FT/IR-6200 instrument. Resonance frequencies are given as wavenumbers in cm^-1^. High resolution mass spectra (HRMS) were recorded on a Bruker maXis (ESI) by the ETH Zürich MS service.

**Synthesis of AX-35 and analogs.** **AX-35** (originally **GW861072X** in Ballell *et al.*, 2013) was synthesized in two steps. EDC-mediated amide coupling of diethylphosphonoacetic acid (**1**) with 1-piperonylpiperazine (**2**) provided phosphonate **3** in 96% yield. Subsequent HWE-reaction with thiophen-2-carbaldehyde (**4i**) afforded **AX-35** in 90% yield (scheme 1). Using intermediate **3** with the corresponding aldehyde in the HWE-reaction, four different analogs, **AX-36** - **AX-39**, were synthesized for initial SAR investigation (scheme 1).

**Scheme 1.** *Reagents and conditions*: (a) EDC, HOBt, Et_3_N, DCM, 80 °C (MW), 3 h, 96%; (b) Aldehydes **4i-v**, NaH, THF, rt, 18 h, 86-98%.

*Diethyl (2-(4-(benzo[d][1,3]dioxol-5-ylmethyl)piperazin-1-yl)-2-oxoethyl)phosphonate* (**3**)

Diethylphosphonoacetic acid (**1**) (434.6 µL, 2.70 mmol, 1.20 eq.), HOBt (365.3 mg, 2.70 mmol, 1.20 eq.), EDC (476.9 µL, 2.70 mmol, 1.20 eq.), Et_3_N (374.6 µL, 2.70 mmol, 1.20 eq.), and 1-piperonylpiperazine (**2**) (496.2 mg, 2.25 mmol, 1.00 eq.) were dissolved in DCM (5.0 mL). The mixture was heated in the microwave at 80 °C for 3 h, quenched with saturated NaHCO_3_ (10 mL) and extracted with DCM (3 x 10 mL). The combined organic layers dried over MgSO_4_, filtered and concentrated in *vacuo*. Flash chromatography (DCM/MeOH 98:2 to 95:5 to 9:1) provided phosphonate **3** (864.1 mg, 2.17 mmol, 96%) as a yellow oil.

**TLC** (SiO_2_; DCM/MeOH 95:5, UV): R*_f_* = 0.16. **^1^H-NMR** (400 MHz, CDCl_3_): δ (ppm) = 6.69 (s, 1H), 6.60 – 6.55 (m, 2H), 5.78 (s, 2H), 4.01 (dq, *J* = 8.2, 7.1 Hz, 4H), 3.52 – 3.39 (m, 4H), 3.27 (s, 2H), 2.91 (d, *J* = 22.0 Hz, 2H), 2.29 (dt, *J* = 31.6, 5.1 Hz, 4H), 1.19 (t, *J* = 7.1 Hz, 6H). **^13^C-NMR** (101 MHz, CDCl_3_): δ (ppm) = 162.8 (d, ^2^*J*_C-P_ = 7.0 Hz), 147.4, 146.5, 131.2, 121.9, 109.0, 107.6, 100.7, 62.3, 62.2 (d, ^2^*J*_C-P_ = 7.2 Hz), 52.6, 52.2, 46.7, 41.7, 33.0 (d, ^1^*J*_C-P_ = 132.8 Hz), 16.1 (d, ^3^*J*_C-P_ = 8.7 Hz). **^31^P-NMR** (162 MHz, CDCl_3_): δ (ppm) = 21.1 (m). **IR** (thin film): ʋ = 2980, 2924, 2813, 1641, 1502, 1489, 1441, 1394, 1367, 1341, 1247, 1139, 1095, 1028, 966, 867, 810, 789, 720, 676, 591, 576 cm^-1^. **HR-MS** (ESI): Calcd for C_18_H_28_N_2_O_6_P [M+H]^+^, 399.1679 *m/z*; Found, 399.1676 *m/z*.

*(E)-1-(4-(Benzo[d][1,3]dioxol-5-ylmethyl)piperazin-1-yl)-3-(thiophen-2-yl)prop-2-en-1-one* (**AX-35**)

To a solution of **3** (753.1 mg, 1.89 mmol, 1.00 eq.) in THF (25.0 mL) were added NaH (54.4 mg, 2.27 mmol, 1.20 eq.) and thiophen-2-carbaldehyde (**4i**) (210.1 µL, 2.27 mmol, 1.20 eq.). The reaction mixture was stirred at rt for 18 h, filtered through celite, then concentrated in *vacuo*. Flash chromatography (DCM/MeOH 98:2 to 95:5) provided **AX-35** (608.5 mg, 1.71 mmol, 90%) as a yellow oil.

**TLC** (SiO_2_; DCM/MeOH 98:2, UV): R*_f_* = 0.22. **^1^H-NMR** (400 MHz, CDCl_3_): δ (ppm) = 7.76 (dt, *J* = 15.1, 0.8 Hz, 1H), 7.25 (dt, *J* = 5.1, 1.0 Hz, 1H), 7.15 (dt, *J* = 3.5, 0.8 Hz, 1H), 6.97 (dd, *J* = 5.1, 3.6 Hz, 1H), 6.83 – 6.80 (m, 1H), 6.72 – 6.67 (m, 2H), 6.63 (d, *J* = 15.1 Hz, 1H), 5.88 (s, 2H), 3.62 (d, *J* = 46.2 Hz, 4H), 3.38 (s, 2H), 2.42 – 2.36 (m, 4H). **^13^C-NMR** (101 MHz, CDCl_3_): δ (ppm) = 165.1, 147.9, 146.9, 140.6, 135.6, 131.7, 130.3, 128.1, 127.2, 122.32, 116.0, 109.5, 108.0, 101.1, 62.7, 62.6, 53.3, 52.7, 45.9, 42.3. **IR** (thin film): ʋ = 3069, 3005, 2895, 2810, 2772, 1638, 1597, 1501, 1488, 1439, 1416, 1367, 1335, 1298, 1282, 1269, 1238, 1205, 1145, 1114, 1096, 1037, 1000, 966, 932, 922, 864, 822, 810, 791, 703, 651, 575, 534, 518, 490 cm^-1^. **HR-MS** (ESI): Calcd for C_19_H_21_N_2_O_3_S [M+H]^+^, 357.1267 *m/z*; Found, 357.1264 *m/z*.

*(E)-1-(4-(Benzo[d][1,3]dioxol-5-ylmethyl)piperazin-1-yl)-3-(thiophen-3-yl)prop-2-en-1-one* (**AX-36**)

To a solution of **3** (1.51 g, 3.79 mmol, 1.00 eq.) in THF (50.0 mL) were added NaH (109.0 mg, 4.54 mmol, 1.20 eq.) and thiophen-3-carbaldehyde (**4ii**) (415.0 µL, 4.54 mmol, 1.20 eq.). The reaction mixture was stirred at rt for 18 h, filtered through celite, then concentrated in *vacuo*. Flash chromatography (DCM/MeOH 98:2 to 95:5) provided **AX-36** (1.30 g, 3.64 mmol, 96%) as a yellow oil.

**TLC** (SiO_2_; DCM/MeOH 95:5, UV): R*_f_* = 0.17. **^1^H-NMR** (400 MHz, CDCl_3_): δ (ppm) = 8.18 (d, *J* = 15.3 Hz, 1H), 7.94 (dd, *J* = 2.8, 1.3 Hz, 1H), 7.83 – 7.78 (m, 2H), 7.37 (d, *J* = 1.3 Hz, 1H), 7.29 – 7.17 (m, 3H), 6.45 (s, 2H), 4.28 – 4.09 (m, 4H), 3.94 (s, 2H), 2.99 – 2.92 (m, 4H). **^13^C-NMR** (101 MHz, CDCl_3_): δ (ppm) = 165.4, 147.7, 146.7, 138.2, 136.3, 131.5, 126.8, 126.7, 125.1, 122.1, 116.7, 109.3, 107.9, 100.9, 62.5, 53.1, 52.6, 45.8, 42.1. **IR** (thin film): ʋ = 3675, 3077, 2987, 2971, 2900, 2811, 2772, 2238, 1731, 1645, 1599, 1502, 1488, 1439, 1412, 1368, 1335, 1298, 1283, 1270, 1239, 1146, 1114, 1094, 1038, 1000, 975, 908, 867, 809, 781, 727, 645, 606, 573, 524, 425 cm^-1^. **HR-MS** (ESI): Calcd for C_19_H_21_N_2_O_3_S [M+H]^+^, 357.1267 *m/z*; Found, 357.1271 *m/z*.

*(E)-1-(4-(Benzo[d][1,3]dioxol-5-ylmethyl)piperazin-1-yl)-3-(1λ^3^,3λ^2^-thiazol-4-yl)prop-2-en-1-one* (**AX-37**)

To a solution of **3** (1.51 g, 3.79 mmol, 1.00 eq.) in THF (50.0 mL) were added NaH (109.2 mg, 4.55 mmol, 1.20 eq.) and thiazole-4-carbaldehyde (**4iii**) (515.0 mg, 4.55 mmol, 1.20 eq.). The reaction mixture was stirred at rt for 18 h, filtered through celite, then concentrated in *vacuo*. Flash chromatography (DCM/MeOH 98:2 to 95:5) provided **AX-37** (1.32 g, 3.70 mmol, 98%) as a yellow oil.

**TLC** (SiO_2_; DCM/MeOH 95:5, UV): R*_f_* = 0.14. **^1^H-NMR** (400 MHz, CDCl_3_): δ (ppm) = 8.83 – 8.74 (m, 1H), 7.64 (dd, *J* = 14.9, 0.8 Hz, 1H), 7.40 (d, *J* = 2.0 Hz, 1H), 7.30 (d, *J* = 15.0 Hz, 1H), 6.84 (d, *J* = 1.3 Hz, 1H), 6.76 – 6.68 (m, 2H), 5.93 (s, 2H), 3.69 (dt, *J* = 28.2, 4.8 Hz, 4H), 3.41 (s, 2H), 2.46 – 2.40 (m, 4H). **^13^C-NMR** (101 MHz, CDCl_3_): δ (ppm) = 165.2, 153.7, 153.6, 153.2, 153.1, 147.7, 146.8, 134.2, 131.5, 122.2, 120.2, 119.8, 109.4, 107.9, 100.9, 62.6, 53.2, 52.6, 45.9, 42.2. **IR** (thin film): ʋ = 3078, 2891, 2811, 2772, 2236, 1646, 1604, 1501, 1488, 1439, 1367, 1334, 1267, 1248, 1238, 1204, 1145, 1114, 1095, 1038, 999, 973, 909, 879, 819, 791, 726, 645, 594, 574, 528, 486 cm^-1^. **HR-MS** (ESI): Calcd for C_18_H_20_N_3_O_3_S [M+H]^+^, 358.1220 *m/z*; Found, 358.1223 *m/z*.

*(E)-1-(4-(Benzo[d][1,3]dioxol-5-ylmethyl)piperazin-1-yl)-3-phenylprop-2-en-1-one* (**AX-38**)

To a solution of **3** (1.50 g, 3.77 mmol, 1.00 eq.) in THF (50.0 mL) were added NaH (108.4 mg, 4.52 mmol, 1.20 eq.) and benzaldehyde (**4iv**) (459.0 µL, 4.52 mmol, 1.20 eq.). The reaction mixture was stirred at rt for 18 h, filtered through celite, then concentrated in *vacuo*. Flash chromatography (DCM/MeOH 98:2 to 95:5) provided **AX-38** (1.24 g, 3.53 mmol, 94%) as a yellow oil.

**TLC** (SiO_2_; DCM/MeOH 95:5, UV): R*_f_* = 0.26. **^1^H-NMR** (400 MHz, CDCl_3_): δ (ppm) = 7.65 (d, *J* = 15.4 Hz, 1H), 7.54 – 7.46 (m, 2H), 7.40 – 7.28 (m, 3H), 6.92 – 6.81 (m, 2H), 6.79 – 6.64 (m, 2H), 5.92 (s, 2H), 3.68 (d, *J* = 39.6 Hz, 4H), 3.42 (s, 2H), 2.53 – 2.36 (m, 4H). **^13^C-NMR** (101 MHz, CDCl_3_): δ (ppm) = 165.4, 147.8, 146.8, 142.7, 135.3, 131.6, 129.6, 128.8, 127.8, 122.2, 117.2, 109.4, 107.9, 101.0, 62.6, 53.2, 52.6, 52.6, 45.9, 42.2. **IR** (thin film): ʋ = 3060, 2810, 2772, 2236, 1646, 1603, 1500, 1488, 1438, 1366, 1334, 1301, 1238, 1205, 1145, 1115, 1096, 1038, 999, 976, 920, 863, 810, 762, 727, 705, 685, 645, 565, 530, 482 cm^-1^. **HR-MS** (ESI): Calcd for C_21_H_23_N_2_O_3_ [M+H]^+^, 351.1703 *m/z*; Found, 351.1707 *m/z*.

*(E)-1-(4-(Benzo[d][1,3]dioxol-5-ylmethyl)piperazin-1-yl)-3-(thiazol-2-yl)prop-2-en-1-one* (**AX-39**)

To a solution of **3** (1.76 g, 4.42 mmol, 1.00 eq.) in THF (50.0 mL) were added NaH (127.2 mg, 5.30 mmol, 1.20 eq.) and thiazole-2-carbaldehyde (**4v**) (465.6 µL, 5.30 mmol, 1.20 eq.). The reaction mixture was stirred at rt for 18 h, filtered through celite, then concentrated in *vacuo*. Flash chromatography (DCM/MeOH 98:2 to 95:5) provided **AX-39** (1.35 g, 3.78 mmol, 86%) as an orange oil.

**TLC** (SiO_2_; DCM/MeOH 95:5, UV): R*_f_* = 0.38. **^1^H-NMR** (400 MHz, CDCl_3_): δ (ppm) = 7.83 (d, *J* = 3.2 Hz, 1H), 7.69 (d, *J* = 15.1 Hz, 1H), 7.36 (d, *J* = 3.2 Hz, 1H), 7.26 (d, *J* = 15.1 Hz, 1H), 6.81 (d, *J* = 1.3 Hz, 1H), 6.72 – 6.67 (m, 2H), 5.90 (s, 2H), 3.65 (dt, *J* = 33.7, 4.9 Hz, 4H), 3.39 (s, 2H), 2.44 – 2.38 (m, 4H). **^13^C-NMR** (101 MHz, CDCl_3_): δ (ppm) = 164.2, 147.9, 146.9, 144.5, 133.5, 131.6, 122.3, 121.7, 121.1, 109.5, 108.1, 101.1, 62.7, 53.3, 52.6, 46.1, 42.5. **IR** (thin film): ʋ = 3448, 3075, 2900, 2811, 2773, 1733, 1643, 1605, 1501, 1487, 1439, 1397, 1367, 1334, 1289, 1238, 1137, 1114, 1094, 1037, 999, 966, 932, 875, 810, 791, 768, 733, 707, 656, 617, 590, 577, 519 cm^-1^. **HR-MS** (ESI): Calcd for C_18_H_20_N_3_O_3_S [M+H]^+^, 358.1220 *m/z*; Found, 358.1227 *m/z*.

# ^1^H-, ^13^C- AND ^31^P-NMR SPECTRA
