# Supplementary material for: Arylvinylpiperazine Amides, a New Class of Potent Inhibitors Targeting QcrB of Mycobacterium tuberculosis
Source: mBio. 2018 Oct 9;9(5):e01276-18. doi: 10.1128/mBio.01276-18 (PMC6178619; doi:10.1128/mBio.01276-18)
Supplement: TABLE S3 [file mbo005184080st3.docx]

# SUPPLEMENTARY TABLES

**Table S3** List of primers used in this study

| Primer name | Sequence |
| --- | --- |
| RvQcrB_S182P_For  (recombineering) | 5’-CTGTCGGGACTCGGTCTGCGCGCGGCACTCTCGCCGATCACGCTGGGTATGCCGGTAATCGGGACCTGGC-3’ |
| RvQcrB_M342V_For  (recombineering) | 5’-ACCATTCCCGCCCCGGTCTGGGTCGCCGTGATCGTGGGCCTGGTTTTCGTCCTGCTACCCGCCTACCCAT-3’ |
| QcrB_full_01F | 5’-AATCCTGTGCCCTTGTCACC-3’ |
| QcrB_full_01R | 5’-AAAATGCGCCGGACTTGAAC-3’ |
| QcrB_full_02F | 5’-CCATCTTGATCCCCAGGCTC-3’ |
| QcrB_full_02R | 5’-AGAAAACTGCCACTACCCGG-3’ |
| QcrB_full_03F | 5’-TGGTTTTCGTCCTGCTACCC-3’ |
| QcrB_full_03R | 5’-GGTGATCGAGTGGCTATACG-3’ |
| cydB_F | 5’-GACGATGCCTACCGATTCGC-3’ |
| cydB_R | 5’-CCAGCCACGTCCAGTCTTTG-3’ |
| lipU_F | 5’-CAAAGGAACACAAGCAGGCG-3’ |
| lipU_R | 5’-GTCTACCTGGTTCCTCGCTG-3’ |
| tgs4_F | 5’-GTCACCTTCGCCAGCATCAA-3’ |
| tgs4_R | 5’-GGTTTGGAGCTCGGTGAATG-3’ |
